# Problematic internet usage in US college students: a pilot study

**DOI:** 10.1186/1741-7015-9-77

**Published:** 2011-06-22

**Authors:** Dimitri A Christakis, Megan M Moreno, Lauren Jelenchick, Mon T Myaing, Chuan Zhou

**Affiliations:** 1 Seattle Children's Research Institute, Center for Child Health, Behavior, and Development2001 Eighth Avenue, Seattle, WA 98121, USA; 2 Department of Pediatrics, University of Washington, 4500 Pacific Street, Seattle, WA 98105, USA; 3 Department of Pediatrics, University of Wisconsin, 2870 University Avenue, Madison, WI 53705, USA

## Abstract

**Background:**

Internet addiction among US college students remains a concern, but robust estimates of its prevalence are lacking.

**Methods:**

We conducted a pilot survey of 307 college students at two US universities. Participants completed the Internet Addiction Test (IAT) as well as the Patient Health Questionnaire. Both are validated measures of problematic Internet usage and depression, respectively. We assessed the association between problematic Internet usage and moderate to severe depression using a modified Poisson regression approach. In addition, we examined the associations between individual items in the IAT and depression.

**Results:**

A total of 224 eligible respondents completed the survey (73% response rate). Overall, 4% of students scored in the occasionally problematic or addicted range on the IAT, and 12% had moderate to severe depression. Endorsement of individual problematic usage items ranged from 1% to 70%. In the regression analysis, depressive symptoms were significantly associated with several individual items. Relative risk could not be estimated for three of the twenty items because of small cell sizes. Of the remaining 17 items, depressive symptoms were significantly associated with 13 of them, and three others had *P *values less than 0.10. There was also a significant association between problematic Internet usage overall and moderate to severe depression (relative risk 24.07, 95% confidence interval 3.95 to 146.69; *P *= 0.001).

**Conclusion:**

The prevalence of problematic Internet usage among US college students is a cause for concern, and potentially requires intervention and treatment amongst the most vulnerable groups. The prevalence reported in this study is lower than that which has been reported in other studies, however the at-risk population is very high and preventative measures are also recommended.

## Introduction

Pathological use of the Internet, whether problematic or truly addictive, remains a growing concern worldwide [1]. Absent formal diagnostic criteria, current approaches model problematic Internet usage on the basis of problematic gambling, extrapolating data from one compulsive, nonpharmacologically addictive behavior to another. Currently, there is no recognized psychiatric diagnosis of Internet addiction, although it is being considered for inclusion in the *Diagnostic and Statistical Manual of Mental Disorders, Fifth Edition *[2].

The prevalence of the problem among US children is not known, but a random digit dial survey of US adults found that as many as one in eight adults are considered addicted [3]. Adolescents and young adults are worthy of special consideration, as they have been shown to be at high risk for behavioral addictions [4]. International estimates of adolescent Internet addiction vary widely. In Europe the prevalence has been reported to be between 1% and 9% [5-9], in the Middle East the prevalence is between 1% and 12% [10-12] and in Asia the prevalence has been reported to be between 2% and 18% [13-20]. However, these data must be interpreted with some caution, as varying scales with questionable validity as well as conflicting reports making accurate estimation difficult. Additionally, the field has been hampered by methodological weaknesses of existing research, with the most salient among them being sampling bias. Many early studies in adults relied on voluntary Internet surveys without measurable denominators, convenience samples of Internet users or chat room sampling [21,22].

College students are a group that may be particularly vulnerable to addiction, as they have largely unfettered, unsupervised access to the Internet and independent control of their time. Estimates of problematic usage in college students vary from 1% to 26% in the US [23-28] and between 6% and 19% internationally [29-31]. Studies in US college students have been limited in several ways, including reliance on a single class for sampling [28] and the use of unvalidated measures or cutoffs [23,26,27,32]. Nevertheless, pediatricians and parents continue to report overuse of the Internet in their patients and children, respectively. Given that the Internet is woven into the fabric of the lives of this generation of children, concerns about the potential for addiction seem warranted and require a systematic estimation of the scope of the problem in a defined population of interest. As part of an ongoing study of college student Internet usage, we collected data on Internet addiction from a representative sample of college students at two large, geographically distinct US universities.

## Methods

The study period was between 1 September 2009 and 15 August 2010, and the protocol was approved by both the University of Wisconsin and the University of Washington Institutional Review Boards.

### Setting and subjects

To gather a target population of Internet users, this study was conducted using the Facebook social networking site (SNS) [33] to identify participants. Facebook was selected because it is the most popular SNS [34-38]. Over 90% of college students use SNSs, and most report daily use. We investigated publicly available Facebook profiles of undergraduate students within two large state university Facebook networks. To be included in the study, profile owners were required to self-report their ages as 18 to 20 years old and provide evidence of profile activity during the past 30 days. We analyzed only profiles for which we could confirm the profile owner's identity by calling a telephone number listed either on the student's Facebook profile or in the university directory.

### Data collection and recruitment

We used the Facebook search engine to identify profiles within our two selected university networks among the freshman, sophomore and junior undergraduate classes. This search yielded 3,038 profiles, all of which were assessed for eligibility. The majority of profiles were ineligible because the profile owners were incorrectly listed and were not undergraduates (*n *= 448), age under 18 years or over 20 years (*n *= 313), no age listed (*n *= 49), no contact information (telephone number or email address) listed on either the Facebook profile or in the university telephone directory (*n *= 303) or because of privacy settings (*n *= 1,630). A total of 307 Facebook profiles met all inclusion criteria, and the profile owners were recruited into this study.

For profiles that met inclusion criteria, profile owners were called on the telephone. After verifying the profile owner's identity, the study was explained to the profile owner and permission was requested to send an email that contained further information about the study. If the participant consented to receive the email, an email was sent to the profile owner's university email account that provided detailed information about the study as well as a link to the online survey. The survey was administered online using a Catalyst WebQ online survey engine. Survey respondents were provided a $15 iTunes gift card as compensation.

### Primary outcome measures

Our primary outcome variable was the Internet Addiction Test (IAT), which has been validated among adults and is used globally [39]. The IAT is composed of 20 questions, with each response measured on a six-point Likert scale (Not at all, Rarely, Occasionally, Often, Always and Does not apply) [40]. Scores of 20 to 49 represent "average" users, scores of 50 to 79 represent "occasional problems" and scores over 80 are classified as "addicted" [40].

Because cultural norms for college students are different from other adults, rendering statements such as "You become defensive when someone asks you what you do online" of questionable validity, we conducted our analyses using both the overall score as well as the IAT's individual items as outcome measures. Although the IAT's individual items have not been independently validated, several have considerable face validity (namely, "You find that you stay online longer than you intended" and "You try to cut down on the amount of time you spend online").

### Covariates

We collected demographic data, including age, race and/or ethnicity and gender, from participating students. In addition, as prior studies have reported associations between depressive symptoms and problematic Internet usage, we had participants complete the Patient Health Questionnaire 9 (PHQ-9), a validated measure of depression in young adults [29,41]. The PHQ-9 categorizes respondents as having "no depression," "minimal symptoms," "moderate depression" or "severe depression."

### Statistical analysis

We scored the IAT according to the proposed algorithm and then dichotomized it as "average user" [42] versus "occasional problems and addict," with the latter defined here as "problematic user." For our analysis of the individual items on the IAT, we dichotomized each one as "frequently" or more. We dichotomized the PHQ-9 as "moderate to severe depression" and used it as a binary predictor. Because relative risk (RR) is a more interpretable summary of association, and because many of our outcomes are not rare so that odds ratios would not approximate RRs, we used a modified Poisson regression approach to estimate RRs [42,43]. This approach for estimating RR on the basis of binary data does not require that the outcome follow a Poisson distribution. For our purposes, the Poisson model was a "working model" to facilitate the estimation and did not affect the consistency of the RR estimation. To remove biases in the standard error estimates, we used model robust sandwich standard error estimates for confidence intervals and hypothesis tests. We adjusted for gender, state, age and race and/or ethnicity. None of these covariates were significant, and for simplicity we therefore presented adjusted RRs for the dichotomized PHQ-9 alone.

## Results

A total of 224 eligible respondents completed the survey (a 73% response rate). The demographic data of our participants are summarized in Table 1. Overall, 4% of students scored in the occasional problem or addicted range on the IAT, and 12% had moderate to severe depression. Endorsement of individual problematic usage items ranged from 1% to 70% (Table 2). In the regression analysis, depressive symptoms were significantly associated with many individual IAT items. RRs could not be calculated for three of the twenty items because of small cell sizes. Of the remaining 17 items, depressive symptoms were significantly associated with 13 of them and three others had *P *values less than 0.10. However, some of the confidence intervals were wide because of the small cell sizes (Table 3). Some of the items worth noting are sleep loss, impact on grades and schoolwork and involvement in household chores. There was also a significant association between moderate to severe depression and problematic Internet usage overall (RR = 24.07, 95% confidence interval 3.95 to 146.69; *P *= 0.001). In other words, students with moderate to severe depression were about 24 times more likely than their peers to exhibit problematic Internet usage.

**Table 1:** Study participant demographics^a^

	Internet usage		
	
Demographic variable	Overall	Average user (*n *= 216)	Problematic user (*n *= 8)
Mean age, yr (range)	18.78 (18 to 20)	18.78 (18 to 20)	18.88 (18 or 19)
Males	46%	48%	25%
Washington State	45%	46%	63%
Race and/or ethnicity			
Caucasian	71%	70%	68%
Asian	18%	18%	25%
Multiracial	8%	8%	0
Other	4%	4%	0
PHQ-9 category			
No depression	56%	58%	13%
Minimal depression symptoms	32%	32%	12%
Moderate depression	9%	7%	63%
Severe depression	3%	3%	12%

^a^PHQ-9, Patient Health Questionnaire 9.

**Table 2:** Summary of individual item response rates from the Internet Addiction Survey

Question	Not applicable	Rarely	Occasionally	Frequently	Often	Always
You find that you stay online longer than you intended	0%	6%	24%	28%	30%	10%
You neglect household chores to spend more time online	2%	28%	45%	13%	9%	2%
You prefer the excitement of the Internet to intimacy with your partner	30%	64%	4%	1%	0%	0%
You form new relationships with fellow online users	12%	62%	20%	4%	0.5%	.5%
Others in your life complain to you about the amount of time you spend online	14%	70%	13%	1%	1%	0%
Your grades or schoolwork suffer because of the amount of time you spend online	7%	50%	33%	6%	3%	1%
You check your email before something else that you need to do	4%	12%	28%	19%	18%	18%
Does your job performance or productivity suffer because of the Internet?	15%	53%	23%	6%	1%	1%
You become defensive or secretive when anyone asks you what you do online	18%	70%	12%	1%	0%	0%
You block out disturbing thoughts about your life with soothing thoughts of the Internet	21%	67%	10%	1%	.5%	.5%
You find yourself anticipating when you will go online again	11%	54%	27%	6%	2%	0%
You fear that life without the Internet would be boring, empty or joyless	15%	55%	21%	7%	1%	0.5%
You snap, yell or act annoyed if someone bothers you while you are online	21%	72%	5%	1%	0%	0%
You lose sleep due to late night logins	11%	50%	25%	9%	5%	0%
You feel preoccupied with the Internet when offline or fantasize about being online	21%	68%	8%	2%	1%	0%
You find yourself saying "Just a few more minutes" when online	7%	31%	33%	17%	7%	4%
You try to cut down the amount of time you spend online	7%	29%	38%	16%	8%	4%
You try to hide how long you've been online	15%	73%	8%	3%	1%	0.5%
You choose to spend more time online than going out with others	15%	74%	8%	2%	0%	0.5%
You feel depressed, moody or nervous when you are offline, which goes away when you are back online	22%	73%	3%	0.5%	1%	0%

**Table 3:** Modified Poisson regression models with individual IAT items as outcome measures and presence of moderate to severe depressive symptoms as primary predictors^a^

Question	RR (95% CI)	*P *value
You find that you stay online longer than you intended	1.31 (1.08 to 1.58)	0.005
You neglect household chores to spend more time online	2.34 (1.34 to 4.10)	0.003
You prefer the excitement of the Internet to intimacy with your partner	11.46 (1.64 to 79.92)	0.014
You form new relationships with fellow online users	3.57 (0.86 to 14.86)	0.08
Others in your life complain to you about the amount of time you spend online	2.24 (0.17 to 29.13)	0.54
Your grades or schoolwork suffer because of the amount of time you spend online	4.69 (1.74 to 12.68)	0.002
You check your email before something else that you need to do	1.31 (0.97 to 1.76)	0.08
Does your job performance or productivity suffer because of the Internet?	4.86 (1.50 to 15.75)	0.008
You become defensive or secretive when anyone asks you what you do online	*	
You block out disturbing thoughts about your life with soothing thoughts of the Internet	*	
You find yourself anticipating when you will go online again	13.18 (4.14 to 41.99)	<0.001
You fear that life without the Internet would be boring, empty or joyless	3.35 (1.03 to 10.88)	0.05
You snap, yell or act annoyed if someone bothers you while you are online	*	
You lose sleep due to late night logins	3.95 (1.92 to 8.10)	<0.001
You feel preoccupied with the Internet when offline or fantasize about being online	11.51 (1.91 to 69.29)	0.008
You find yourself saying "Just a few more minutes" when online	1.79 (1.14 to 3.41)	0.01
You try to cut down the amount of time you spend online	1.94 (1.11 to 2.81)	0.02
You try to hide how long you've been online	41.57 (3.79 to 455.35)	0.002
You choose to spend more time online than going out with others	10.54 (0.94 to 118.30)	0.06
You feel depressed, moody or nervous when you are offline, which goes away when you are back online	31.71 (2.82 to 356.01)	0.005

^a^RR, relative risk; 95% CI, 95% confidence interval; *inestimable based on cell size.

## Discussion

In a representative sample from two large state universities, we found that the prevalence of problematic Internet usage was 4%. To put the prevalence in perspective, if it is confirmed, problematic Internet usage would be as common as asthma in a similar population of children [44]. Beyond problematic usage, it is clear that many college students have concerns about their Internet usage with respect to other relevant domains in their lives. In particular, the fact that 70% reported that they stay online longer than they intend suggests that the ubiquity and ease of access to the Internet are not without a potential downside. Our current understanding of addictions suggests that some people are at greater risk than others based on genetic predisposition [43]. Whether these susceptible individuals actually develop an addiction involves many factors, but repeated exposure to the substrate is clearly necessary, be it to alcohol or to gambling. If we extrapolate that there is, in a similar way, an inherent susceptibility to Internet addiction, then today's college students are clearly at risk, given the considerable exposure that they have to the Internet and the high prevalence of self-expressed concerns about their reliance on it [45].

These findings advance our understanding of Internet addiction by improving upon previous study methodologies in several ways. First, our sampling method targeted college students from two geographically distinct universities and sampled them in their entirety. Given that up to 98% of college students have an SNS profile and that most report daily use, our approach has considerable generalizability [35,36,38]. Second, we corroborated an association between depressive symptoms and problematic Internet usage that has been found in international samples [13,46]. This association lends further validity to the phenomenon of Internet addiction, as depression has been associated with other behavioral addictions, such as gambling [48].

There are several limitations to this study that warrant mention. First, the cross-sectional nature of the analytic plan precludes drawing causal inferences about the association between depressive symptoms and problematic Internet usage. However, others have found similar associations using longitudinal study designs [49]. We suggest that depression and problematic Internet usage are linked in a mutually enhancing cycle wherein depression begets social isolation, which begets problematic Internet usage and thus increases both social isolation and depression. Second, our sample size was modest, making robust estimates difficult, but this does not diminish the statistical significance of our findings. Moreover, on the basis of our findings in an ongoing systematic review of the existing literature that we performed [50], the present study comprises the largest sample of US college students that has used a validated instrument of Internet addiction and that has presented prevalence data on problematic Internet use. Third, many of our potential subjects were excluded because of privacy settings. The extent to which this might bias our findings is unclear. Facebook has updated and changed privacy setting options over the past few years, leaving many Facebook users confused and even angry at the complexity of the currently available settings [51-53]. People who are more familiar with Facebook may have been more likely to adjust privacy settings and more likely to exhibit problematic usage, potentially biasing our estimate downward, but this remains an empirical question. Finally, although the scale we used has been validated in adults, it may not be ideally suited to the adolescent or college student population. A distinct measure of problematic Internet usage is clearly needed for these age groups. In the interim, our estimate is probably conservative, given the nature of some of the questions asked. Furthermore, the individual domain analysis suggests that there is the potential for significant problematic usage among college students.

In spite of these limitations, the results of this pilot study have some important implications for college students and administrators. Problematic Internet usage is prevalent on US college campuses. Colleges should consider both preventative approaches in the form of awareness campaigns leveraging students' self-reported concerns about their usage of the Internet, and, for some students, treatment might even be warranted. Finally, given that Internet usage begins during early to middle childhood, pediatricians should make assessment of Internet usage a part of preventative practice [54].

## Authors' contributions

DAC and MMM conceived of and designed the study. LJ administered the survey and collected the data. MTM and CZ were in charge of data analysis and were assisted by DAC and MMM. DAC, MMM and LJ drafted the manuscript. All authors reviewed and approved the final version of the manuscript.

## Pre-publication history

The pre-publication history for this paper can be accessed here:

http://www.biomedcentral.com/1741-7015/9/77/prepub
